# Diabetic foot ulcer with osteomyelitis, successfully treated with the holistic approach of multiple ayurvedic treatment modalities - A case report

**DOI:** 10.1016/j.ijscr.2023.108315

**Published:** 2023-05-12

**Authors:** Swapna Bopparathi, Narasimha Raju K.V

**Affiliations:** Associate Professor, Department of Shalya Tantra, National Institute of Ayurveda, deemed to be University, Amer Road, Jaipur, Rajasthan, India; Professor, Department of Kaya Chikitsa, Jyoti Vidyapeeth Ayurvedic University, Jaipur, Rajasthan, India

**Keywords:** Amputation, Case report, Diabetic foot, Hirudo medicinalis, Osteomyelitis, Triphala

## Abstract

**Introduction:**

Diabetic foot ulcer (DFU) with osteomyelitis is the devastating condition, which is a challenge to surgeons in saving the limb of the patient and in many circumstances ends up with amputation, which leaves physical and psychosocial trauma for both the patient and patient's family.

**Presentation of case:**

A 48-year-old female patient with uncontrolled type 2 diabetes presented with swelling and gangrenous deep circular ulcer of size approx. 3 × 4 cm on plantar aspect of great toe of her left foot with involvement of first webspace from last three months. Plain X ray showed disrupted and necrotic proximal phalanx suggestive of diabetic foot ulcer with osteomyelitis. Despite using antibiotics and antidiabetic drugs for past three months she didn't get significant response and was suggested for toe amputaion. Hence, she approached our hospital for further treatment. We successfully treated the patient with the holistic approach of surgical debridement, medicinal leech therapy (MLT), irrigation of the wound with triphala decoction, jatyadi tail dressings, oral ayurvedic antidiabetic drugs to control blood sugar levels and a mixture of herbo mineral drug which is having antimicrobial property.

**Discussion:**

DFU may lead to infection, gangrene, amputation, death of the patient. Hence it is the need of the hour to look for limb salvage treatment modalities.

**Conclusion:**

The holistic approach of these ayurvedic treatment modalities are effective and safe in treating DFUs with osteomyelitis and in preventing amputation.

## Highlights

• Osteomyelitis complicated DFU frequently ends up with amputation.
• Amputations cause impaired physical function and psychosocial trauma.
• Majority of DFUs don't respond to standard treatment.
• Interdisciplinary care and multimodal treatments are needed.
• Multiple ayurvedic treatments such as surgical debridement, MLT etc. can treat DFU.

## 1 Introduction

A frequent metabolic illness that has a serious impact on public health is Diabetes mellitus (DM) [1]. One of the more significant consequences of diabetes is diabetic foot ulcer (DFU), result of a number of pathogenic conditions, including neuropathy and peripheral vascular disease. Additionally, the ulcers are more vulnerable to infection due to the compromised immune system and diminished microcirculation [1,2]. This soft tissue infection causes diabetic foot osteomyelitis (DFO), which is present in 10 %–15 % of moderate and 50 % of severe infections. It progresses into the bone by first affecting the cortex and later the marrow [3]. These osteomyelitis complicated ulcers frequently require surgical interventions, long term antibiotic therapy [4] and amputation which increase prolonged hospitalization and rate of mortality [5].

Numerous conventional therapies are available for managing DFUs, including “antibiotic therapy, surgical debridement, wound dressing, hyperbaric oxygen therapy (HBOT), negative pressure wound therapy (NPWT), stem cell-based therapy, growth factor therapy, and maggot debridement therapy (MDT)” [6,7]. Amputation is the most common clinical result, occurring in 6/1000 patients annually, and the lifetime incidence of foot ulceration in the diabetic community is as high as 15 %. Males are three times more likely to acquire DFUs than females [[8], [9], [10]]. “Amputations have diverse impacts on patients, including impaired physical function, psychosocial trauma, loss of employment and economic stress” [11].

To overcome with all the negative effects of amputation, limb saving treatment modalities are required where integration with other system of medicine is necessary for the benefit of the patient, where in multiple ayurvedic treatment modalities such as medicinal leech therapy (MLT), cleansing with triphala decoction, jatyadi tail dressings and oral ayurvedic medicines etc. can play a major role. Here, we successfully treated a case of DFU with osteomyelitis in our hospital, an academic Institution by using said treatment modalities and this case report has followed the SCARE criteria [12].

## 2 Presentation of case

A 48-year-old female patient with uncontrolled type 2 diabetes presented to outdoor unit of our hospital on 18th January 2017, with swelling and gangrenous ulcer on great toe of her left foot from last three months. She was brought to the hospital in a wheelchair. She is a home maker from a middle level of socioeconomic status and her BMI is 26.7 kg/m^2^ (overweight). As per the history given by patient, she was asymptomatic four months back and was diagnosed with type 2 diabetes and started using antidiabetic drugs but not on a regular basis. Three months back, in the month of October 2016, she had a blunt trauma on her left great toe which developed into an ulcer and didn't show any tendency towards healing with antibiotics & antidiabetic drugs and over the next two months the swelling gradually increased.

She has been using an antidiabetic compound drug composed of glipizide 5 mg with metformin 500 mg, morning and evening with meal from last four months to control blood sugar levels. She used multiple oral antibiotics such as amoxycillin trihydrate 500 mg with clavulanate potassium 125 mg for 20 days, three times a day (TID) after meals, ciprofloxacin 500 mg, BD for 20 days and had no surgical intervention for the current clinical condition. She is not a smoker and denied for drug and alcohol abuse. Patient doesn't have any family history of diabetes mellitus.

On physical examination of the foot all the peripheral arterial pulsations were normal and edema in first four toes, extended up to transverse arch of the sole on ventral aspect and mid of the foot on dorsal aspect with gangrenous less painful circular, Grade 3 deep ulcer (Wagner classification) of size 3 × 4 cm on plantar aspect of great toe with the involvement of first web space (Fig. A). The floor of the ulcer was covered with necrosed tissue, purulent and foul smelled discharge. After obtaining written informed consent patient was admitted in our hospital and the investigation reports on admission and during treatment were as follows (Table 1).

**Fig. A:**
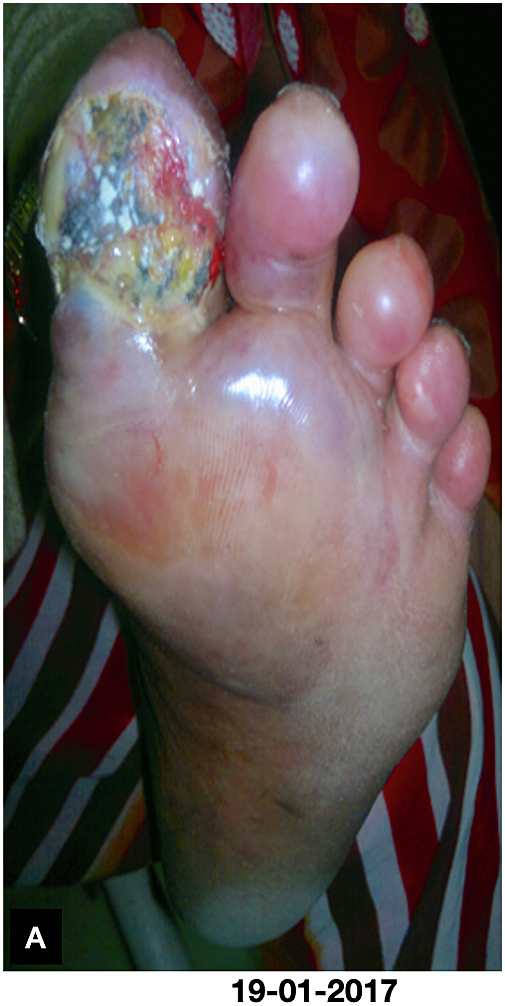
Image captured on first day of visit,

**Table 1:** The investigation reports of the patient on admission, during treatment and ten days before discharge from hospital.

S. no.	Investigation	Normal range	Values on date			
			18/01/2017	20/01/2017	31/01/2017	15/02/2017
1	Haemoglobin	11–16 %	12.7 %	–	11.8 %	12.8 %
2	Total Leucocyte Count (TLC)	4.0–10 th/uL	7400/uL	–	8700/uL	6200/uL
3	Erythrocyte Sedimentation Rate (ESR)	0–20 mm/h	62 mm/h	–	64 mm/h	10 mm/h
4	Neutrophils	50–70 %	61 %	–	85 %	55 %
5	Lymphocytes	20–40 %	32 %	–	35 %	15 %
6	Monocytes	3.0–12.0 %	05 %	–	03 %	02 %
7	Eosinophils	0.5–5 %	02 %	–	01 %	01 %
8	Total platelet count (TPLC)	1.00–3.00 lakhs/UL	3.69 lakhs/UL	–	2.63 lakhs/UL	1.82 lakhs/UL
9	Random Blood Sugar (RBS)	90–140 mg%	115 mg%	–	–	–
10	SGOT	<37 IU/l	22 IU/l	–	18 IU/l	–
11	SGPT	<42 IU/l	29 IU/l	–	19 IU/l	–
12	Alkaline Phosphatase (ALP)	64–306 IU/l	196 IU/l	–	271 IU/l	–
13	Fasting blood sugar (FBS)	60–110 mg%	–	99 mg%	369 mg%	101 mg%
14	Postprandial blood sugar (PPBS)	<140 mg%	–	142 mg%	455 mg%	138 mg%
15	Serum C- Reactive Protein (CRP)	–	–	–	Positive	–

**Fig. B1, B2:**
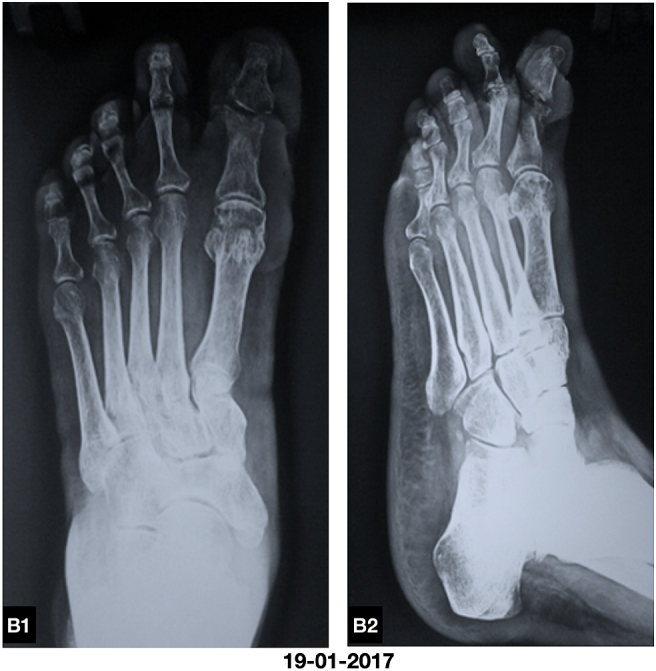
Plain X ray showed disrupted cortex and lytic lesions of the distal phalanx and head of proximal phalanx of great toe with soft tissue swelling.

Plain X ray of the foot showed disrupted cortex and lytic lesions of the distal phalanx and head of proximal phalanx of great toe with soft tissue swelling (Fig. B1, B2). The diagnosis was made as diabetic foot ulcer with osteomyelitis, on the clinical ground and plain X ray of the foot.

Patient didn't get relief with the conventional treatment methods and was suggested for amputation but the patient was not willing for it and approached our hospital for further treatment.

We started the treatment with removal of necrosed and infectious tissue by surgical debridement on 22nd January 2017 and was done by the first author. The ulcer was treated with a combination therapy including medicinal leech therapy, cleansing the wound with triphala decoction and dressing with jatyadi tail. For leech therapy, *Hirudo medicinalis* was used weekly once for two months (eight sessions), in every session three leeches were used, after disinfecting them in saline water and approximately 12 to 15 ml of blood used to be sucked out by the leeches in each session. Bloodletting with leeches removes the inflammatory mediators from wound site and fresh oxygenated blood will perfuse the area with which healing will be promoted. The saliva of leech is with full of antimicrobial, anti-inflammatory enzymes which reduce the bacterial load and promotes wound healing. Cleansing with *triphala* decoction and topical application of *jatyadi tail* were continued till the healing of the wound and bone. All these procedures were done by the trained nursing staff. Blood sugar levels were controlled with a mixture of ayurvedic antidiabetic drugs, *madhumehari churna* 5 g, *prameha prahari churna* 1 g, 12th hourly, oral administration on empty stomach with water for three months. As this is a case of antibiotic resistant DFO, a mixture of herbomineral drugs which has antimicrobial property was suggested to consume orally, such as *ashtamurthy rasayan* 250 mg, *guduchi satwa* 1 g and *amlaki churna* 3 g, 12th hourly on empty stomach with honey for 45 days. During the treatment complete offloading of the foot and limb elevation was done along with a diet plan, in which restricted the quantity of carbohydrate intake to one roti (made of barley flour) or a bowl of dhaliya (made of broken barley) with a medium size bowl of cooked vegetables and daal (pulses) in each meal, two meals a day.

**Fig. C1, C2, D1, D2, E:**
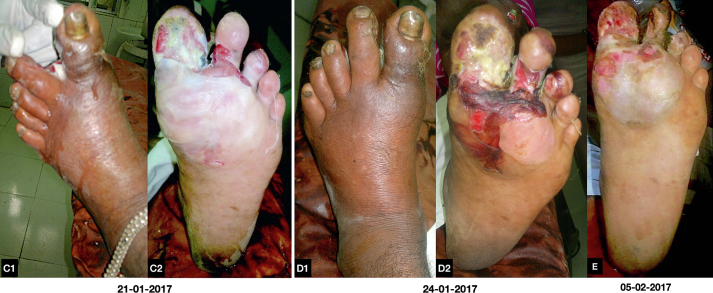
Serial photographs during treatment.

**Fig. G1, G2:**
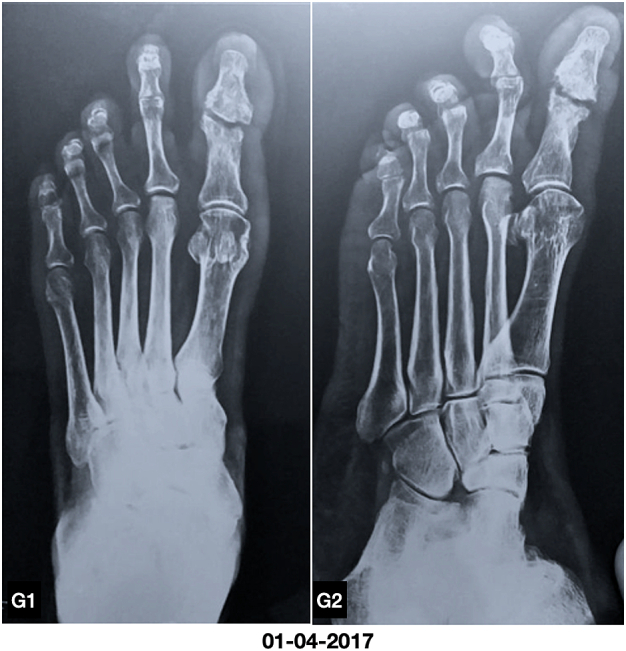
Plain X ray showed completely healed disrupted and necrosed distal and proximal phalanges of great toe with periosteal new bone.

Following treatment, blood sugar levels (FBS-101 mg%), CRP, and ESR all returned to normal. Within two months of beginning treatment, the toes erythema, edema had all gradually disappeared and ulcers were partially healed (Fig. C1, C2, D1, D2, E, Fig. F). Patient was discharged from the hospital on 28th February 2017 with a good health condition and was instructed regarding the care of the wound to avoid pressure on the limb, offloading, be on walking aid or wheelchair till complete recovery, diet plan and suggested for follow up on every week. Complete healing of the wound and bone was achieved in two months ten days. A plain radiograph revealed completely healed disrupted and necrosed distal and proximal phalanges of great toe with periosteal new bone (Fig. G1, G2). The medication is well tolerated and has no local or systemic adverse events. After full recovery, the patient was kept on the same ayurvedic anti-diabetic medication at the same dosage along with the same diet plan that was prescribed during hospitalization for the following six months. After then, no diet plan was followed; only medication was used. After three years of follow up patient had no sign of recurrence and for these three years, she was taking the prescribed medication to keep her blood glucose levels under control, and every three to six months, she had her blood sugar levels checked and results were within the normal range.

**Fig. F:**
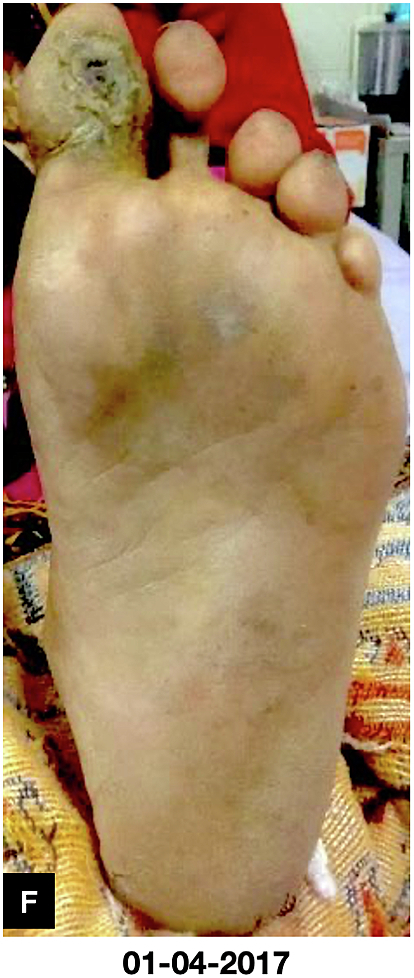
Complete healing of wound and bone about 2 and half months after treatment initiation;

Patient is ecstatic with the outcome of this ayurvedic treatment. Patient claimed that “I was scared of losing my toe and now I am quite happy with my complete recovery. I felt very comfortable with every therapeutic modality which rendered complete healing.”

## 3 Discussion

DFU may lead to infection, gangrene, amputation, death of the patient. The three cornerstones of DFU with osteomyelitis management are debridement, offloading, and infection control [13]. However, only 30 % of DFUs recover in 20 weeks with standard treatment methods [14] and monotherapy approach would result in a relatively low level of recovery because there are numerous pathogenic pathways that produce DFUs. As a result, interdisciplinary care and multimodal care are needed for DFU management [15].

In ayurveda, non- healing chronic ulcers were incorporated under the clinical entity Dushta vrana [16] where in different types, symptomatology, management was explained in detail; MLT [17,18], cleansing with medicated decoction etc. are among those various treatment modalities. The cumulative effect of these treatments had yielded tremendous wound healing. MLT's therapeutic use was proven in many clinical studies to heal diabetic foot ulcers [19,20]. As, ischemia is one of the causes of DFU, MLT augments blood flow by vasodilation (because of histamine in leech saliva) with which inflammatory material and bacterial load will be reduced and wound healing enhances. More than 20 known bioactive compounds, including antistasin, eglins, guamerin, hirudin, saratin, bdellins, complement, and carboxypeptidase inhibitors, are secreted by leeches. They feature analgesic, anti-inflammatory, platelet inhibitory, anticoagulant, and thrombin regulating properties in addition to extracellular matrix degrading and antimicrobial effects [21] which ultimately aid in painless procedure.

Jatyadi tail, used for dressing of the wound has Picchrorhiza kurroa and purified blue vitriol (CuSO4) as ingredients which have proven wound healing property. Picchrorhiza kurroa [22] (Katukarohini) increases angiogenesis, epithelialization and migration of endothelial cells, dermal myoblasts, and fibroblasts into the wound bed. Purified blue vitriol [23] (Tuttha) (CuSO4) promotes Vascular Endothelial Growth Factor (VEGF) expression in the wound.

Cleansing with triphala decoction; *Triphala,* the fruits of three medicinal plants; *Terminalia chebula* Retz. (*Hareetaki*), Terminalia bellerica Roxb. (*Vibheetaki*) and Emblica officinalis Gaertn. (*Amlaki*), is considered as most versatile of all herbal formulations had proven antibacterial, antiviral, antifungal, antihelmintic, antimutagenic and anticarcinogenic properties [24]. As this case was resistant to antibiotics, mixture of ayurvedic oral drugs which are having antimicrobial property, ashtamurthy rasayan, amlaki rasayan and guduchi satwa were used to control infection. In our case, the cumulative effect of said multimodal treatments had shown significant results with complete healing of the wound and bone and there was no sign of recurrence even after three years of follow up.

## 4 Conclusion

In diabetic foot ulcer with osteomyelitis, surgery is frequently required to remove the infected and necrosed soft tissues and bone. But with holistic approach of these ayurvedic multimodal treatments complete healing of the ulcer and bone can be achieved in selected cases and this case shows the successful healing of the ulcer and bone in less than three months. Patient tolerance and satisfaction score are high without any local or systemic adverse reactions and the drugs are well accepted by the patients. Hence these complete therapeutic procedures are highly recommended to cut costs and improve wound healing because treating DFU is an expensive and time-consuming process.

## Consent

Written informed consent was obtained from the patient for publication of this case report and accompanying images. A copy of the written consent is available for review by the Editor-in-Chief of this journal on request.

## Ethical approval

Case reports are exempted from ethical approval in our institute, National Institute of Ayurveda Hospital, Jaipur, Rajasthan, India.

## Funding

None.

## CRediT authorship contribution statement

**Dr. Swapna Bopparathi:** contributed in care of this patient, conceptualization, design, acquisition, analysis and interpretation of data, and drafting this case report.

**Dr. Narasimha Raju. K.V:** contributed in design of the study, review and revising the report critically.

Both authors approved the final version of manuscript.

## Guarantor

Dr. Swapna Bopparathi.

## Declaration of competing interest

None.
